# Surrogate Markers of Cardiovascular Risk and Chronic Obstructive Pulmonary Disease: A Large Case-Controlled Study

**DOI:** 10.1161/HYPERTENSIONAHA.117.10151

**Published:** 2018-08-20

**Authors:** Marie Fisk, Carmel M. McEniery, Nichola Gale, Kaisa Mäki-Petäjä, Julia R. Forman, Margaret Munnery, Jean Woodcock-Smith, Joseph Cheriyan, Divya Mohan, Jonathan Fuld, Ruth Tal-Singer, Michael I. Polkey, John R. Cockcroft, Ian B. Wilkinson

**Affiliations:** From the Division of Experimental Medicine and Immunotherapeutics, University of Cambridge, United Kingdom (M.F., C.M.M., K.M.-P., J.W.-S., J.C., I.B.W.); School of Healthcare Sciences (N.G.) and Department of Cardiology, Wales Heart Research Institute (M.M., J.R.C.), Cardiff University, United Kingdom; Cambridge Clinical Trials Unit (J.R.F., J.C., I.B.W.) and Division of Respiratory Medicine (J.F.), Cambridge University Hospitals NHS Foundation Trust, United Kingdom; GSK R&D, King of Prussia, PA (D.M., R.T.S.); and NIHR Respiratory Biomedical Research Unit, Royal Brompton & Harefield NHS Foundation Trust and Imperial College, London, United Kingdom (D.M., M.I.P.).

**Keywords:** cardiovascular diseases, carotid intima-media thickness, case-control studies, pulmonary disease, chronic obstructive, pulse wave analysis

## Abstract

Cardiovascular disease is a common comorbidity and cause of mortality in chronic obstructive pulmonary disease. A better understanding of mechanisms of cardiovascular risk in chronic obstructive pulmonary disease patients is needed to improve clinical outcomes. We hypothesized that such patients have increased arterial stiffness, wave reflections, and subclinical atherosclerosis compared with controls and that these findings would be independent of smoking status and other confounding factors. A total of 458 patients with a diagnosis of chronic obstructive pulmonary disease and 1657 controls (43% were current or ex-smokers) with no airflow limitation were matched for age, sex, and body mass index. All individuals underwent assessments of carotid–femoral (aortic) pulse wave velocity, augmentation index, and carotid intima–media thickness. The mean age of the cohort was 67±8 years and 58% were men. Patients with chronic obstructive pulmonary disease had increased aortic pulse wave velocity (9.95±2.54 versus 9.27±2.41 m/s; *P*<0.001), augmentation index (28±10% versus 25±10%; *P*<0.001), and carotid intima–media thickness (0.83±0.19 versus 0.74±0.14 mm; *P*<0.001) compared with controls. Chronic obstructive pulmonary disease was associated with increased levels of each vascular biomarker independently of physiological confounders, smoking, and other cardiovascular risk factors. In this large case-controlled study, chronic obstructive pulmonary disease was associated with increased arterial stiffness, wave reflections, and subclinical atherosclerosis, independently of traditional cardiovascular risk factors. These findings suggest that the cardiovascular burden observed in this condition may be mediated through these mechanisms and supports the concept that chronic obstructive pulmonary disease is an independent risk factor for cardiovascular disease.

## Abstract 2

Supplemental Digital Content is available in the text.

**See Editorial Commentary, pp 409–410**

Chronic obstructive pulmonary disease (COPD) is a common, complex condition that is a worldwide leading cause of death, disability, and poor health.^1^ Although primarily a lung disease, extrapulmonary manifestations contribute to poor health and are associated with increased risk of mortality and hospitalization.^2^ Cardiovascular disease (CVD) is one of the most common comorbidities observed in COPD. Indeed, a third of all deaths in patients with COPD are from CVD, and patients with COPD have 2× to 5× increased risk of CVD compared with the general population.^3,4^ Whether shared risk factors, such as smoking, fully explain the association is currently unclear. Therefore, a better understanding of cardiovascular risk in patients with COPD and the underlying mechanisms is needed to improve clinical outcomes.

Previous studies have reported inconsistent data on surrogate biomarkers of cardiovascular risk observed in patients with COPD. A major source of variation between studies is how cardiovascular risk is evaluated, inherent differences between populations, variable adjustment for confounders, and small sample sizes.^5^ Several small studies have examined aortic pulse wave velocity (aPWV), the gold standard measure of arterial stiffness and an independent predictor of cardiovascular events and mortality in the general population,^6^ and consistently report elevated aPWV in patients with COPD.^7^ However, it is less clear whether this association is simply related to smoking. In contrast, in the Copenhagen Heart Study, augmentation index (AIx), a composite measure of arterial wave reflections and arterial stiffness, was increased in subjects with airflow obstruction, but this was considered to be because of smoking and not a direct complication of COPD.^8^ Fewer studies have examined carotid intima–media thickness (CIMT), a marker of subclinical atherosclerosis, in COPD, although 1 study reported smokers with mild airflow limitation to have increased CIMT compared with smokers without airflow limitation.^9^ However, as far as we are aware, comprehensive assessments of these vascular biomarkers have not previously been undertaken in the same group of patients with COPD or with appropriate adjustment for confounding variables.

We hypothesized that patients with COPD compared with age-, sex-, and body mass index (BMI)–matched controls have elevated arterial stiffness, increased wave reflections, and increased subclinical atherosclerosis, independently of smoking and other confounding factors. We also sought to evaluate both the impact of smoking and presence of COPD per se on these vascular biomarkers and the relationship between these vascular biomarkers and COPD severity.^10^

## Methods

Data and analysis methods that support the findings of this study are available from the corresponding author on reasonable requests. Study materials and methods used to conduct the study are described in detail below. ERICA (Evaluation of the Role of Inflammation in Chronic Airways disease) is a prospective, observational study of patients with COPD recruited from 5 UK centers.^11^ The study was approved by the Cambridge South Research Ethics Committee (REC: 11/EE/0357), and all participants provided written informed consent. The study was performed in accordance with institutional guidelines and in accordance with the Declaration of Helsinki. For this analysis, we included data from patients with COPD recruited from the Cambridge and Cardiff study sites only because controls from the ACCT (Anglo Cardiff Collaborative Trial) had also been studied at both sites. Expanded methods are provided in the online-only Data Supplement.

### Patients With COPD

Patients were aged ≥40 years, with ≥10 pack years smoking history, a clinical diagnosis of COPD, and postbronchodilator forced expiratory lung volume in 1 second <80% and forced expiratory lung volume in 1 second/forced vital capacity ratio <0.7. Patients had to be clinically stable and free of exacerbations in the preceding 4 weeks before enrollment in the study.

### Control Subjects

Controls were selected in a frequency-matched manner from an anonymized database of the ACCT study^12^ to provide 3 age-, sex-, and BMI-matched controls for every ERICA patient with COPD using bands of continuous variables of age and BMI. Only subjects with smoking status recorded were included in the analysis, and subjects with a diagnosis of COPD, or spirometry that would support a diagnosis of COPD (forced expiratory lung volume in 1 second/forced vital capacity <0.7), were excluded. Besides these demographics, all other variables were unselected and blinded during the matching process, including all hemodynamic data, vascular biomarkers of interest, cardiovascular risk factors, CVD, and other disease history.

### Vascular Biomarkers and Laboratory Assessments

In all individuals, blood pressure was recorded after 15 minutes of seated rest using a validated osillometric device (HEM 750CP; Omron Corporation, Japan). Radial artery waveforms were then recorded using a high fidelity micromanometer (SPC-301; Millar Instruments) and SphygmoCor software (AtCor Medical, Australia), which generated a corresponding central (ascending aortic) pressure waveform, using a validated transfer function.^13^ From this, the AIx, a measure of wave reflections, was calculated. After a further period of supine rest, blood pressure was reassessed and then carotid–femoral aPWV, a measure of aortic stiffness, calculated using the SphygmoCor device by sequential recording of ECG-gated pressure waveforms at the carotid and femoral sites, as previously described, with surface distances measured using a tape measure.^14^

High-resolution B-mode ultrasound was used to determine CIMT of the common carotid arteries, measured 1 cm from the bulb. Image analysis was performed using Vascular Tools 5 software (Medical Imaging Application PLC), with the larger of the 2 values (left or right) used in analysis. CIMT measurement was available for only a limited number of controls (n=279). Further information on assessments is provided in the online-only Data Supplement.

### COPD Severity

Patients with COPD were classified into quartiles of the BODE Index (BMI [B], degree of airflow obstruction [O], and functional dyspnea [D], and exercise capacity [E] as assessed by the 6-minute walk test). score,^10^ which is a multidimensional tool of COPD severity, that predicts mortality in COPD (see online-only Data Supplement). Vascular biomarkers were compared across quartiles. In addition, the relationship of exacerbations with vascular biomarkers was examined.

### Statistical Analysis

Study size was calculated to obtain at least 3 control subjects for every patient with COPD to allow precise quantification of any potential differences in patients with COPD versus controls. This approach was undertaken because previous smaller case-controlled studies have assessed only 1 vascular biomarker using a 1:1 approach and often not adjusted for physiological confounders.^7^ Data were analyzed using SPSS (v23), and a *P*<0.05 was deemed significant for statistical analyses. Student *t* test, χ^2^ test and general linear models with covariates were used to evaluate differences between groups, with adjustment for study site performed in all analyses, and adjustments made for known physiological confounders of vascular biomarkers if statistically different between groups. For aPWV, adjustments for heart rate, mean arterial pressure, age, sex, and BMI were made if required. For AIx, adjustments for heart rate, age, sex, and height, and for CIMT, adjustments for age and systolic blood pressure were similarly performed if required. To determine the impact of smoking, we evaluated vascular biomarkers stratified by smoking status within each subject group and compared patients with COPD to specifically current smoking/ex-smoker controls. To determine whether COPD was associated with each vascular biomarker, data from patients and controls were combined, and COPD diagnosis (yes/no) binary coded and included as an independent variable in regression analyses. Furthermore, odds ratios were calculated for aPWV >10 m/s and CIMT >0.90 mm, as thresholds defined for increased vascular risk,^15^ adjusted for variables associated with each vascular biomarker. Data are expressed as means±SD or percentages. Bar graphs represent mean values, and error bars represent 95% confidence intervals.

## Results

The demographic characteristics of the patients with COPD and controls are shown in Table 1. Data from 2115 subjects (458 patients, 1657 controls) were available. There were no differences between the patients and controls for age, sex, or BMI. As expected, the proportion of current and ex-smokers in the COPD group versus controls was significantly higher (*P*<0.001). A total of 18 (3.9%) patients with COPD used supplementary oxygen. Cardiovascular comorbidity was increased in patients compared with controls. Use of antihypertensive therapy was similar between groups, but more patients with COPD were taking cholesterol-reducing treatments (*P*<0.001 versus controls).

**Table 1.**
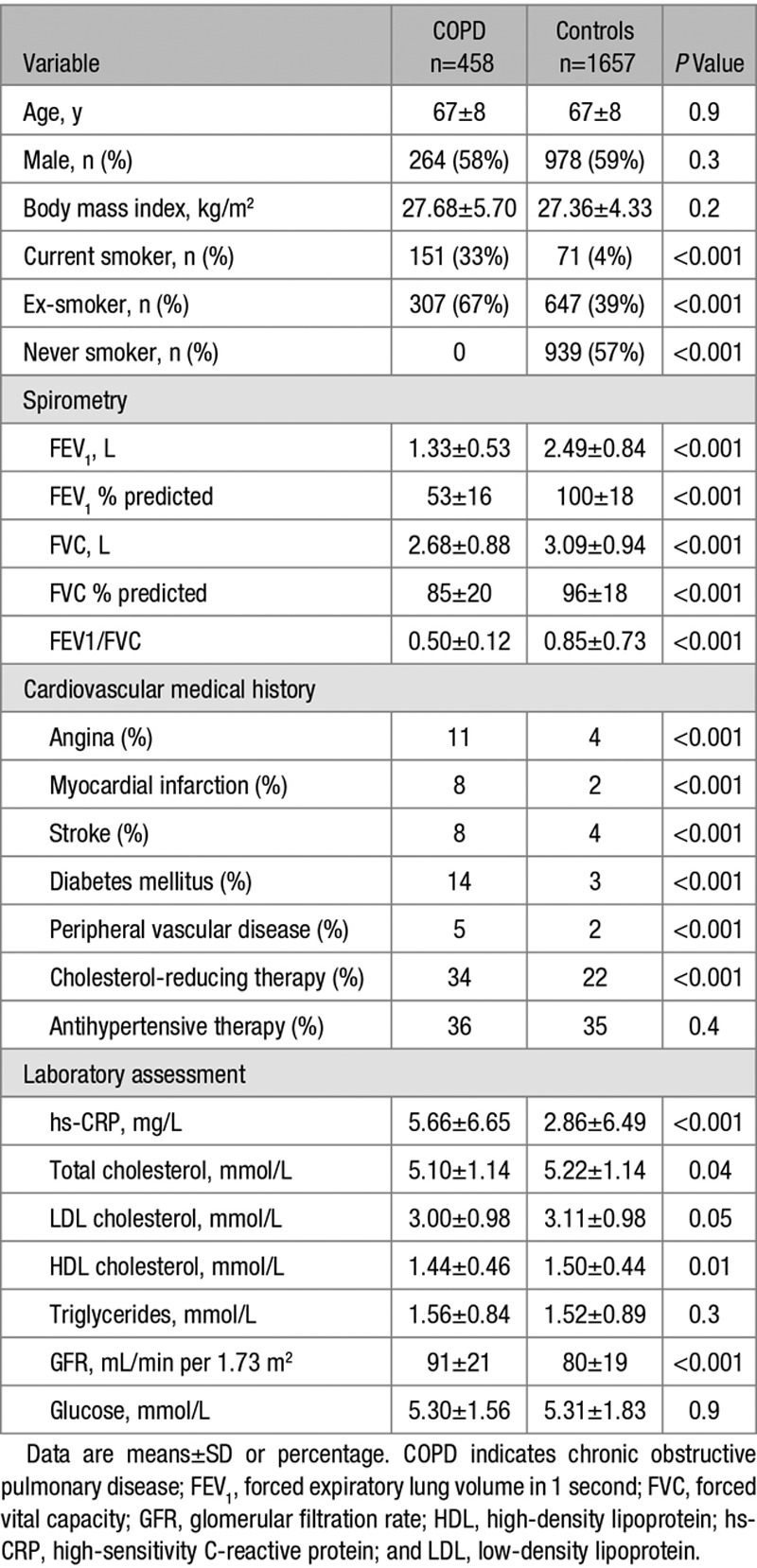
Demographic Characteristics of Patients With COPD and Controls

### Vascular Biomarkers

Data are summarized in Table 2. Systolic blood pressure and heart rate were higher in patients with COPD compared with controls (*P*<0.001 for both). In addition, aPWV, AIx, and CIMT were all higher in patients with COPD, even after adjustment for confounders (*P*<0.001 for all; Figure). Similar trends were observed when patients with COPD were compared with control smokers/ex-smokers only (Table 3) and when individuals from both groups without known cardiovascular comorbidity were compared (Table S1 in the online-only Data Supplement). Analysis of AIx in subjects aged ≤55 years was performed because age-related changes in AIx are more prominent in younger individuals, and AIx is known to plateau after 55 years of age.^12,16^ This showed a mean difference of +10% AIx in patients with COPD versus controls (25±11% versus 15±10%; *P*<0.001), adjusted for heart rate, height, and study site. In contrast, above this age cutoff and in the whole-study data set, smaller +3% differences (*P*<0.001 for both) in AIx were observed between patients with COPD and controls.

**Table 2.**
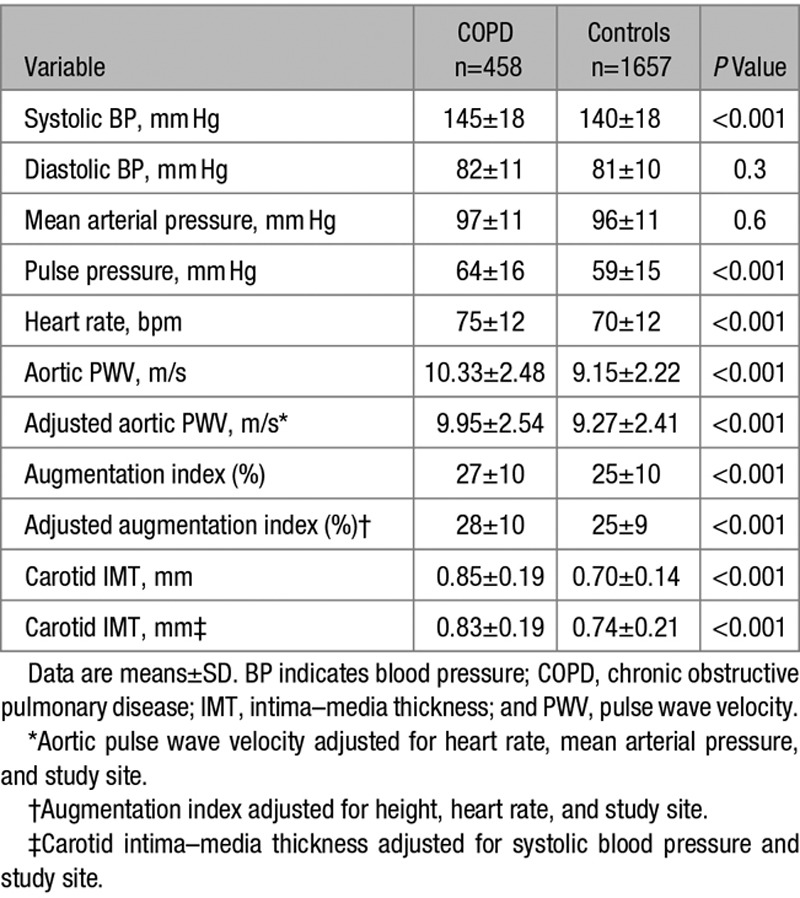
Hemodynamic Characteristics of Patients With COPD and Controls

**Table 3.**
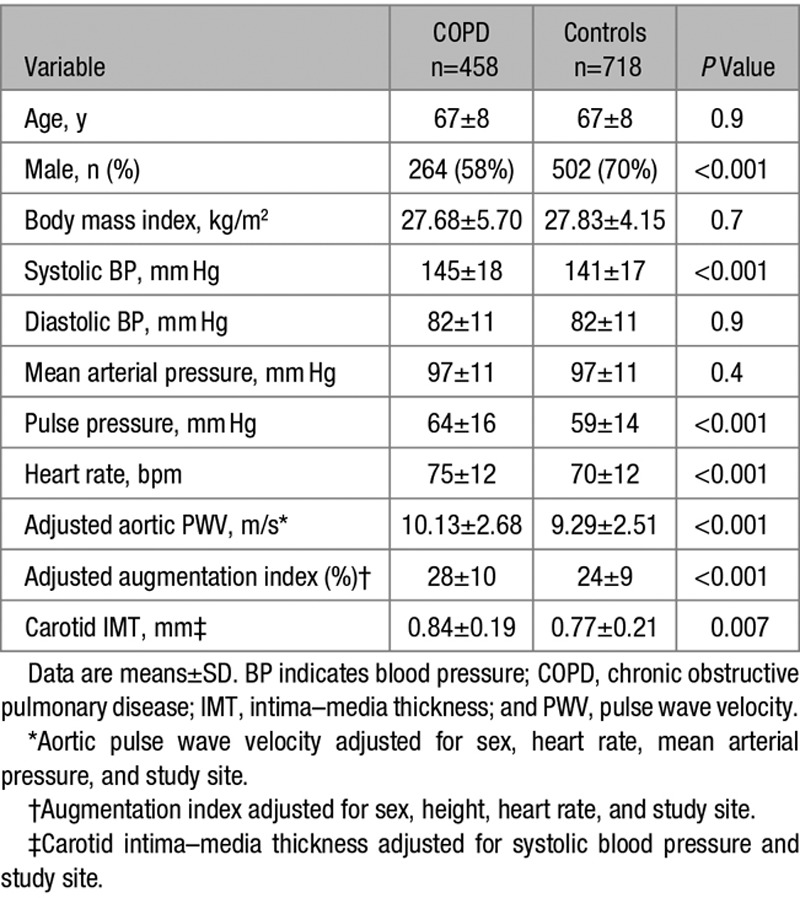
Demographic and Hemodynamic Characteristics of Patients With COPD and Controls Who Are Current/Ex-Smokers

**Figure.**
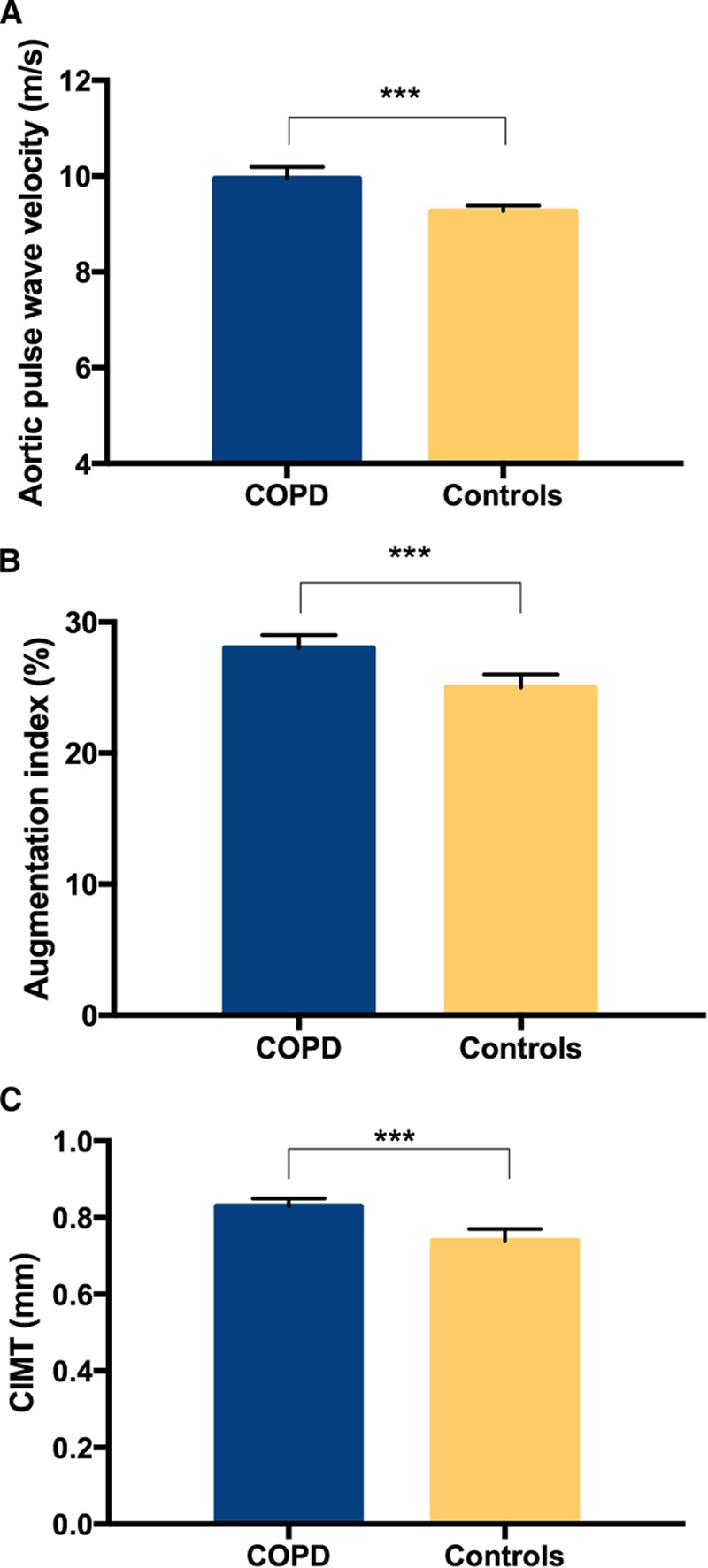
Comparison of surrogate cardiovascular risk markers (**A**) aortic pulse wave velocity, (**B**) augmentation index, and (**C**) carotid intima–media thickness (CIMT) between patients with chronic obstructive pulmonary disease (COPD) and controls. Bars represent mean values, error bars represent 95% confidence intervals. Aortic pulse wave velocity is adjusted for heart rate, mean arterial pressure, and study site. Augmentation index is adjusted for height, heart rate, and study site. CIMT is adjusted for systolic blood pressure and study site. ***P<0.001.

### Impact of Smoking

Table S2 shows demographic and vascular biomarkers stratified by smoking status in patients with COPD and controls. There were no significant differences in aPWV or AIx in controls when stratified by smoking status. The small number of CIMT measurements in controls did not permit a subanalysis of CIMT by smoking status. In patients with COPD, AIx was elevated in current versus ex-smokers, but both COPD current and ex-smokers had increased AIx compared with controls (29±7% versus 25±9%, *P*<0.001 and 27±7% versus 25±9%, *P*<0.001, respectively). Furthermore, there were no differences in aPWV or CIMT in patients with COPD according to smoking status. In patients with COPD, total pack years smoked (47±27 years) was not associated with aPWV, AIx, or CIMT after adjustment for physiological confounders.

### Impact of COPD

A diagnosis of COPD was positively associated with aPWV, AIx, and CIMT, and the associations remained after adjustment for physiological confounders and cardiovascular risk factors (Table 4). Moreover, patients with COPD were 2× to 5× more likely to have increased cardiovascular risk defined by thresholds of aPWV >10 m/s and CIMT >0.90 mm,^15^ odds ratio: 2.29 (95% confidence interval: 1.74, 3.01) and odds ratio: 5.83 (95% confidence interval: 3.15, 10.77) respectively, after adjustment for those variables remaining independently associated with each vascular biomarker in regression analyses.

**Table 4.**
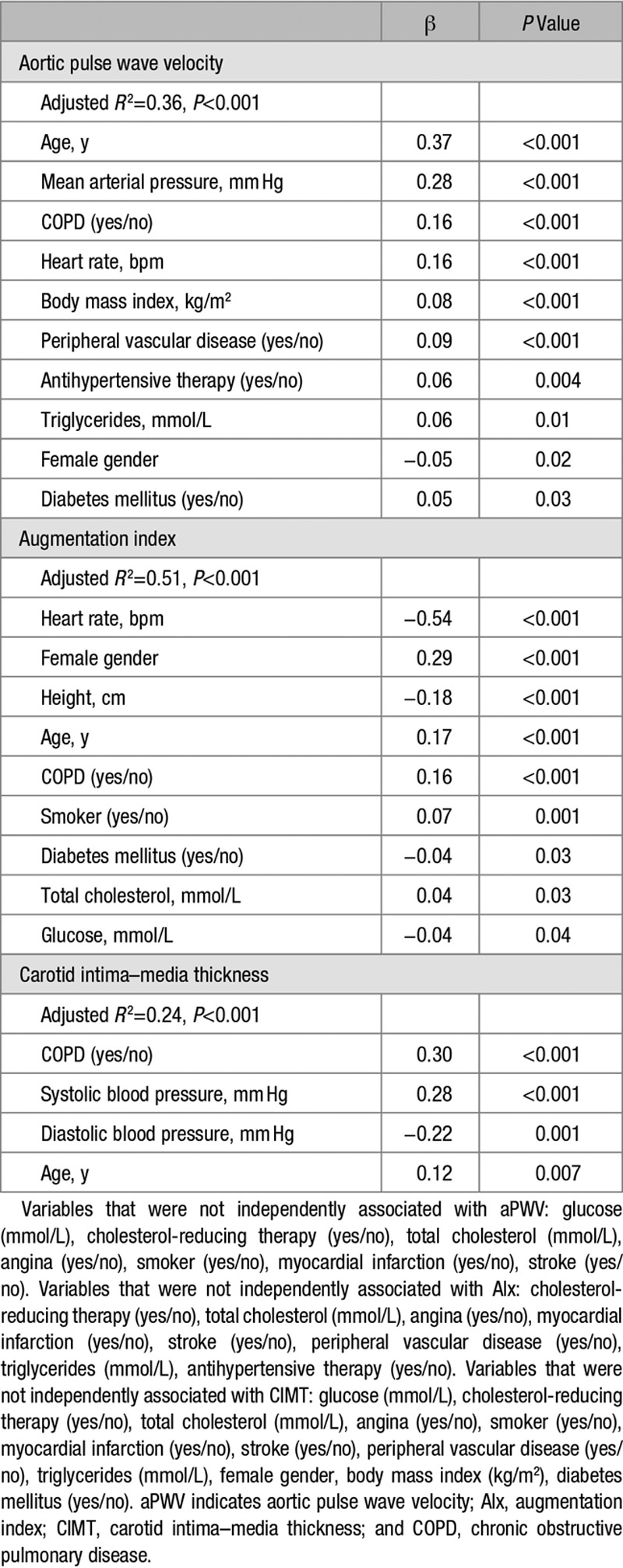
Variables Associated With aPWV, AIx, and CIMT

### COPD Severity and Vascular Biomarkers

A total of 427 patients with COPD had sufficient data to classify into BODE Index quartiles, Table S3. Patients with COPD in the fourth quartile had higher aPWV than all other quartiles (Figure S1), even after adjustment for respiratory medication and peripheral oxygen saturations differences, but in contrast, AIx and CIMT were similar across quartiles. Furthermore, the BODE Index score was positively associated with aPWV, independently of physiological confounders and cardiovascular risk factors, Table S4. However, no associations between exacerbations and vascular biomarkers were observed.

## Discussion

We have shown that surrogate markers of cardiovascular risk are increased in patients with COPD compared with age-, sex-, and BMI-matched controls, independently of known cardiovascular risk factors, including smoking, and physiological confounders. In patients with COPD, a positive relationship between disease severity and specifically arterial stiffness was observed but not for wave reflections or subclinical atherosclerosis. Interestingly, smoking quantified by total pack years smoked was not independently associated with vascular biomarkers within patients with COPD.

Aortic stiffness, defined by aPWV, was +0.7 m/s higher in patients with COPD compared with controls. This is a smaller difference than previously reported,^7^ which may be because of our much larger group of unselected controls, and full adjustment for physiological confounders. Nevertheless, it is still a clinically important difference, suggesting accelerated vascular aging in patients with COPD, because a recent meta-analysis reported that a +1-m/s difference in aPWV is associated with ≈15% increased risk of cardiovascular events and mortality.^17^ Importantly, we have demonstrated that the presence of COPD was independently associated with aPWV and that patients with COPD were twice as likely as controls to have increased arterial stiffness, even after accounting for the potential influence of confounding factors in a fully adjusted model. The lack of difference in aPWV in different groups of patients with COPD stratified by smoking status together with the lack of any independent association between aPWV and total pack years smoked is in keeping with published data in COPD.^18^

For the first time, we have demonstrated that a diagnosis of COPD had a stronger impact on AIx than did smoking and other cardiovascular risk factors. Also, the larger difference in AIx in those patients with COPD versus controls aged ≤55 years is likely to be clinically important because it suggests increased cardiovascular risk even in younger patients with COPD.^12^ In a previous smaller study, Mills et al^19^ observed an increase in augmentation pressure rather than AIx in patients with COPD compared with healthy controls who were not on any regular medication and had no other comorbidities, matched for smoking. Janner et al^8^ examined a general population and found that subjects with obstructive spirometry had higher AIx, but, crucially, smoking confounded this association. Although patients with COPD who were current smokers had increased AIx compared with COPD ex-smokers in our study, COPD per se was associated with increased AIx compared with controls, irrespective of smoking status, and there was no relationship with total pack years smoked. A further consideration is that AIx is influenced by dynamic changes in peripheral vascular resistance. We did not assess resistance in our study, but Casiglia et al^20^ found that patients with COPD versus controls had increased calf blood flow evaluated by venous occlusion plethysmography, as well as lower leg vascular resistance, but did not report total peripheral resistance. If systemic resistance was lower in our patients with COPD, this would be expected to reduce AIx, therefore reducing the observed difference between them and controls.

Further novel findings of the current study include greater CIMT in patients with COPD versus controls, together with the independent association between COPD and higher CIMT, and our finding that patients with COPD are 5× more likely than controls to have increased CIMT, independent of confounding factors. The Kuopio Ischaemic Heart Disease Study indicates that even a +0.1-mm increase in CIMT is associated with +11% risk of myocardial infarction, suggesting that the differences between patients and controls seen in our study are likely to be clinically important.^21^ In addition, in patients with COPD in the current study, CIMT was similar between current and ex-smokers, and there was no association between CIMT and total pack years smoked. Taken together, these observations suggest that the presence of COPD indicates a vulnerability to smoke injury rather than magnitude of smoke exposure.

There are few previous studies of CIMT relevant to COPD to enable direct comparison with our data. In the MESA study (Multi-Ethnic Study of Atherosclerosis), CIMT was associated with decrements in forced expiratory lung volume in 1 second.^22^ In a study of 200 patients with COPD, CIMT >0.9 mm and cardiac infarction injury score >20 were used to define cardiovascular comorbidity, but the study did not include controls or patients with CIMT ≤0.9 mm.^23^ Interestingly, common genetic loci implicated in determining lung function also influence CIMT,^24^ suggesting that shared genetic factors may influence susceptibility to lung and vascular damage.

The independent association between the presence of COPD and arterial stiffness, wave reflections, and CIMT implies that COPD should be considered an independent cardiovascular risk factor, and further studies are necessary to robustly calculate its predictive value for cardiovascular events. This is in keeping with findings by Finkelstein et al^25^ who reported that COPD increased the likelihood of CVD, adjusted for comorbidities and socioeconomic factors including tobacco use. Interestingly, in the current study, a higher proportion of patients with COPD than controls reported taking cholesterol-reducing therapies (reflected in their lower lipid profile levels), suggesting that consideration of cardiovascular risk reduction in patients with COPD had already occurred. Patients with COPD also had higher glomerular filtration rate values although this likely represents lower fat-free muscle mass for equivalent BMI, rather than better renal function.^26^ However, patients with COPD had increased systolic blood pressure compared with controls despite comparable rates of antihypertensive therapy between groups. Assuming equivalent compliance, this suggests that more intense treatment of systolic blood pressure may be required in patients with COPD to achieve the same reduction in blood pressure compared with controls and is an area that requires further research. Patients with COPD also had a higher prevalence of self-reported cardiovascular comorbidities compared with controls. However, prevalence may be even higher when assessed objectively. For example, peripheral vascular disease prevalence was 8.8% when assessed by ankle–brachial index in patients with COPD in a recent European cohort (compared with 5% self-reported in our study) although two thirds of these subjects did not report peripheral vascular disease in their medical history.^27^

Why we observed a relationship between COPD severity defined by the BODE Index^10^ and aPWV, but not AIx, or CIMT highlights the different nature of these vascular parameters. Indeed, the novel association between aPWV and the BODE Index score suggests a complex interaction between vascular stiffness with the score’s composite variables of lung function, symptoms, and physical capacity. BODE may also enhance cardiovascular risk stratification in patients with COPD because its relationship with aPWV was independent of traditional cardiovascular risk factors. One potential explanation for the relationship between COPD severity and specifically aPWV is a shared pathological mechanism of degradation of elastin fibers from the lungs and large arteries because elastin is known to confer elasticity to both organs. A previous study showed that patients with COPD had excessive cutaneous elastin loss, which related to increased cutaneous elastolytic activity, emphysema severity, and arterial stiffness.^28^ Therefore, the possibility of a systemic elastin degradation pathology contributing to the pulmonary and vascular features associated with COPD is raised^28^ although other mechanisms, such as the effects of hypoxia, may also be important. Notably, an inverse association between peripheral oxygen saturations and aPWV in patients with COPD has been observed.^29^ In contrast to aPWV, AIx is a composite measure of wave reflections and arterial stiffness, and CIMT is a complex measure of subclinical atherosclerosis determined by intima thickening and medial hypertrophy driven by pulse pressure. These 2 vascular markers may be elevated in patients with COPD but may not be mechanistically linked to the severity of COPD itself and do not add value to the Framingham risk score, unlike aPWV.^6,30^

The strengths of our study are its size, inclusion of controls that were not selected to be healthy, and adjustment for known physiological confounders and established cardiovascular risk factors. Moreover, we included a high percentage of smokers and ex-smokers in our control group, emphasizing the influence of COPD per se on these vascular biomarkers. The main limitation is its cross-sectional nature, which does not permit any assessment of causality. A further limitation is that we were unable to adjust for factors that were not measured, such as physical activity levels, or those unique to patients with COPD, such as respiratory medications. The potential influence on our results of medications, such as inhaled β-agonists, should be considered. β-agonists are associated with increased risk of cardiovascular events,^31^ but they also reduce wave reflections, mediated, in part, by their effect on the nitric oxide pathway.^32^ The long-term influence of such medications on the vascular health of patients with COPD is thus difficult to gauge, but trial data have showed no beneficial effect on aPWV, of 6-month treatment of combined inhaled corticosteroid and long-acting β-agonist versus placebo.^33^

## Perspectives

We have demonstrated that patients with COPD compared with controls have elevated arterial stiffness, wave reflections, and subclinical atherosclerosis. Moreover, the presence of COPD was associated with aPWV, AIx, and CIMT, independently of traditional cardiovascular risk factors, and arterial stiffness measured by aPWV was positively associated with COPD severity, defined by the BODE Index score. These data support the notion that the presence of COPD is an independent risk factor for CVD, and further studies are needed to determine the impact of the presence of COPD in vascular risk models. In addition, because these vascular biomarkers are plausible mechanisms mediating increased cardiovascular risk in COPD, trials of novel therapies targeted toward these surrogate risk markers are required.

## Appendix

ERICA Consortium: Charlotte Bolton, Peter Calverley, Joseph Cheriyan, John Cockcroft, Marie Fisk, Julia Forman, Jonathan Fuld, Nichola Gale, David Lomas, William MacNee, Mellone Marchong, Carmel McEniery, Bruce Miller, Divya Mohan, Sridevi Nagarajan, Michael Polkey, Ruth Tal-Singer, Ian Wilkinson.

The ACCT Study Investigators: John Cockcroft, Zahid Dhakam, Stacey Hickson, Julia Howard, Kaisa Maki-Petaja, Barry McDonnell, Carmel McEniery, Karen Miles, Maggie Munnery, Pawan Pusalkar, Christopher Retallick, Jane Smith, Edna Thomas, Sharon Wallace, Ian Wilkinson, Susannah Williams, Jean Woodcock-Smith, Yasmin.

## Acknowledgments

We thank all individuals who took part in this study. M. Fisk, C.M. McEniery, I.B. Wilkinson, J.R. Cockcroft, J. Cheriyan, M.I. Polkey, D. Mohan, and R. Tal-Singer contributed to the conception and study design. M. Fisk, C.M. McEniery, N. Gale, J. Woodcock-Smith, M. Munnery, and J. Fuld contributed to the acquisition of data. M. Fisk, C.M. McEniery, K. Mäki-Petäjä, and J.R. Forman contributed to data analysis. We acknowledge Ali B.A.K Hadithi for his help with data analysis. All authors contributed to data interpretation, drafting, and revising the manuscript and have approved the final version for publication. All authors are accountable for all aspects of the work performed.

## Sources of Funding

This study was funded, in part, by a grant from Innovate UK as a component study of the ERICA (Evaluation of the Role of Inflammation in Chronic Airways disease) Consortium. GlaxoSmithKline (GSK), a consortium partner, made contributions toward study management. The work was also funded, in part, by the British Heart Foundation (Project Grant PG/11/35/28879) and the National Institute of Health Research Cambridge Biomedical Research Centre.

## Disclosures

M. Fisk and I.B. Wilkinson received an educational award from GSK during the study period, GSK provide compensation for 50% of J. Cheriyan’s National Health Service salary for clinical trial work, J. Fuld reports personal fees from GSK outside this submitted work, J.R. Cockcroft received a grant from GSK outside this submitted work, M.I. Polkey reports grants from GSK and Innovate UK during the conduct of this study, and D. Mohan and R. Tal-Singer are employees and shareholders of GSK. The other authors report no conflicts.

## Supplementary Material

## Novelty and Significance

### What Is New?

- We have shown for the first time that patients with chronic obstructive pulmonary disease (COPD) have elevated levels of arterial stiffness, wave reflections, and subclinical atherosclerosis independently of potential confounders, including smoking. Furthermore, arterial stiffness was associated with COPD severity, defined by the BODE Index (body mass index [B], degree of airflow obstruction [O], and functional dyspnea [D], and exercise capacity [E] as assessed by the 6-minute walk test) score.

### What Is Relevant?

- Patients with COPD have stiffer arteries, increased subclinical atherosclerosis, and higher systolic blood pressure compared with age-, gender-, body mass index–matched controls despite more patients with COPD being on cholesterol-reducing therapies and equivalent rates of antihypertensive therapies between groups.

### Summary

This large case-controlled study showed that surrogate markers of cardiovascular risk were increased in patients with COPD compared with controls independently of confounding factors.

## References

[1] Mannino DM, Braman S (2007). The epidemiology and economics of chronic obstructive pulmonary disease.. Proc Am Thorac Soc.

[2] Decramer M, Janssens W (2013). Chronic obstructive pulmonary disease and comorbidities.. Lancet Respir Med.

[3] McGarvey LP, John M, Anderson JA, Zvarich M, Wise RA, TORCH Clinical Endpoint Committee (2007). Ascertainment of cause-specific mortality in COPD: operations of the TORCH Clinical Endpoint Committee.. Thorax.

[4] Chen W, Thomas J, Sadatsafavi M, FitzGerald JM (2015). Risk of cardiovascular comorbidity in patients with chronic obstructive pulmonary disease: a systematic review and meta-analysis.. Lancet Respir Med.

[5] Cinarka H, Kayhan S, Gumus A, Durakoglugil ME, Erdogan T, Ezberci I, Yavuz A, Ozkaya S, Sahin U (2014). Arterial stiffness measured via carotid femoral pulse wave velocity is associated with disease severity in COPD.. Respir Care.

[6] Ben-Shlomo Y, Spears M, Boustred C (2014). Aortic pulse wave velocity improves cardiovascular event prediction: an individual participant meta-analysis of prospective observational data from 17,635 subjects.. J Am Coll Cardiol.

[7] Vivodtzev I, Tamisier R, Baguet JP, Borel JC, Levy P, Pépin JL (2014). Arterial stiffness in COPD.. Chest.

[8] Janner JH, McAllister DA, Godtfredsen NS, Prescott E, Vestbo J (2012). Is chronic obstructive pulmonary disease associated with increased arterial stiffness?. Respir Med.

[9] Iwamoto H, Yokoyama A, Kitahara Y, Ishikawa N, Haruta Y, Yamane K, Hattori N, Hara H, Kohno N (2009). Airflow limitation in smokers is associated with subclinical atherosclerosis.. Am J Respir Crit Care Med.

[10] Celli BR, Cote CG, Marin JM, Casanova C, Montes de Oca M, Mendez RA, Pinto Plata V, Cabral HJ (2004). The body-mass index, airflow obstruction, dyspnea, and exercise capacity index in chronic obstructive pulmonary disease.. N Engl J Med.

[11] Mohan D, Gale NS, McEniery CM, Bolton CE, Cockcroft JR, MacNee W, Fuld J, Lomas DA, Calverley PM, Shale DJ, Miller BE, Wilkinson IB, Tal-Singer R, Polkey MI, ERICA Consortium (2014). Evaluating the role of inflammation in chronic airways disease: the ERICA study.. COPD.

[12] McEniery CM, Yasmin, Hall IR, Qasem A, Wilkinson IB, Cockcroft JR, ACCT Investigators (2005). Normal vascular aging: differential effects on wave reflection and aortic pulse wave velocity: the Anglo-Cardiff Collaborative Trial (ACCT).. J Am Coll Cardiol.

[13] Pauca AL, O’Rourke MF, Kon ND (2001). Prospective evaluation of a method for estimating ascending aortic pressure from the radial artery pressure waveform.. Hypertension.

[14] Wilkinson IB, Fuchs SA, Jansen IM, Spratt JC, Murray GD, Cockcroft JR, Webb DJ (1998). Reproducibility of pulse wave velocity and augmentation index measured by pulse wave analysis.. J Hypertens.

[15] ESH/ESC Task Force for the Management of Arterial Hypertension (2013). 2013 Practice guidelines for the management of arterial hypertension of the European Society of Hypertension (ESH) and the European Society of Cardiology (ESC): ESH/ESC Task Force for the Management of Arterial Hypertension.. J Hypertens.

[16] Fantin F, Mattocks A, Bulpitt CJ, Banya W, Rajkumar C (2007). Is augmentation index a good measure of vascular stiffness in the elderly?. Age Ageing.

[17] Vlachopoulos C, Aznaouridis K, Stefanadis C (2010). Prediction of cardiovascular events and all-cause mortality with arterial stiffness: a systematic review and meta-analysis.. J Am Coll Cardiol.

[18] Sabit R, Bolton CE, Edwards PH, Pettit RJ, Evans WD, McEniery CM, Wilkinson IB, Cockcroft JR, Shale DJ (2007). Arterial stiffness and osteoporosis in chronic obstructive pulmonary disease.. Am J Respir Crit Care Med.

[19] Mills NL, Miller JJ, Anand A, Robinson SD, Frazer GA, Anderson D, Breen L, Wilkinson IB, McEniery CM, Donaldson K, Newby DE, Macnee W (2008). Increased arterial stiffness in patients with chronic obstructive pulmonary disease: a mechanism for increased cardiovascular risk.. Thorax.

[20] Casiglia E, Pavan L, Marcato L, Leopardi M, Pizziol A, Salvador P, Zuin R, Pessina AC (1998). Subjects with obstructive pulmonary disease tend to be chronically vasodilated.. Clin Sci (Lond).

[21] Salonen JT, Salonen R (1993). Ultrasound B-mode imaging in observational studies of atherosclerotic progression.. Circulation.

[22] Barr RG, Ahmed FS, Carr JJ, Hoffman EA, Jiang R, Kawut SM, Watson K (2012). Subclinical atherosclerosis, airflow obstruction and emphysema: the MESA Lung Study.. Eur Respir J.

[23] Vanfleteren LE, Spruit MA, Groenen M, Gaffron S, van Empel VP, Bruijnzeel PL, Rutten EP, Op ‘t Roodt J, Wouters EF, Franssen FM (2013). Clusters of comorbidities based on validated objective measurements and systemic inflammation in patients with chronic obstructive pulmonary disease.. Am J Respir Crit Care Med.

[24] Sabater-Lleal M, Mälarstig A, Folkersen L (2014). Common genetic determinants of lung function, subclinical atherosclerosis and risk of coronary artery disease.. PLoS One.

[25] Finkelstein J, Cha E, Scharf SM (2009). Chronic obstructive pulmonary disease as an independent risk factor for cardiovascular morbidity.. Int J Chron Obstruct Pulmon Dis.

[26] Rutten EPA, Bakke PS, Pillai SG, Wagers S, Grydeland TB, Gulsvik A, Wouters EFM (2012). The association between body composition and self-reported co-morbidity in subjects with chronic obstructive pulmonary disease.. OJIM.

[27] Houben-Wilke S, Jörres RA, Bals R (2017). Peripheral artery disease and its clinical relevance in patients with chronic obstructive pulmonary disease in the COPD and Systemic Consequences-Comorbidities Network Study.. Am J Respir Crit Care Med.

[28] Maclay JD, McAllister DA, Rabinovich R, Haq I, Maxwell S, Hartland S, Connell M, Murchison JT, van Beek EJ, Gray RD, Mills NL, Macnee W (2012). Systemic elastin degradation in chronic obstructive pulmonary disease.. Thorax.

[29] McAllister DA, Maclay JD, Mills NL, Mair G, Miller J, Anderson D, Newby DE, Murchison JT, Macnee W (2007). Arterial stiffness is independently associated with emphysema severity in patients with chronic obstructive pulmonary disease.. Am J Respir Crit Care Med.

[30] Bots ML, Groenewegen KA, Anderson TJ (2014). Common carotid intima-media thickness measurements do not improve cardiovascular risk prediction in individuals with elevated blood pressure: the USE-IMT collaboration.. Hypertension.

[31] Salpeter SR, Ormiston TM, Salpeter EE (2004). Cardiovascular effects of beta-agonists in patients with asthma and COPD: a meta-analysis.. Chest.

[32] Wilkinson IB, Hall IR, MacCallum H, Mackenzie IS, McEniery CM, van der Arend BJ, Shu YE, MacKay LS, Webb DJ, Cockcroft JR (2002). Pulse-wave analysis: clinical evaluation of a noninvasive, widely applicable method for assessing endothelial function.. Arterioscler Thromb Vasc Biol.

[33] Bhatt SP, Dransfield MT, Cockcroft JR, Wang-Jairaj J, Midwinter DA, Rubin DB, Scott-Wilson CA, Crim C (2017). A randomized trial of once-daily fluticasone furoate/vilanterol or vilanterol versus placebo to determine effects on arterial stiffness in COPD.. Int J Chron Obstruct Pulmon Dis.

