# Supplementary material for: Surrogate Markers of Cardiovascular Risk and Chronic Obstructive Pulmonary Disease: A Large Case-Controlled Study
Source: Hypertension. 2018 Aug 20;71(3):499–506. doi: 10.1161/HYPERTENSIONAHA.117.10151 (PMC5805278; doi:10.1161/HYPERTENSIONAHA.117.10151)
Supplement: Supplementary file 1 [file hyp-71-499-s001.doc]

**ONLINE SUPPLEMENT**

**Surrogate Markers of Cardiovascular Risk and Chronic Obstructive Pulmonary Disease: A Large Case-Controlled Study**

**Authors:** Marie Fisk PhD^1*^, Carmel M McEniery PhD^1*^, Nichola Gale PhD^2^, Kaisa Mäki-Petäjä PhD^1^, Julia R Forman PhD^3^, Margaret Munnery^4^, Jean Woodcock-Smith^1^, Joseph Cheriyan FRCP^1,3^, Divya Mohan PhD^5,7^, Jonathan Fuld PhD^6^, Ruth Tal-Singer PhD^5^, Michael I Polkey PhD^7^, John R Cockcroft FRCP^4^, Ian B Wilkinson DM^1,3^, on behalf of the ERICA Consortium and ACCT Investigators

1. Division of Experimental Medicine and Immunotherapeutics, University of Cambridge, Cambridge, UK
2. School of Healthcare Sciences, Cardiff University, Cardiff, UK
3. Cambridge Clinical Trials Unit, Cambridge University Hospitals NHS Foundation Trust, Cambridge, UK
4. Department of Cardiology, Wales Heart Research Institute, Cardiff University, Cardiff, UK
5. GSK R&D, King of Prussia, Pennsylvania, USA
6. Division of Respiratory Medicine, Cambridge University Hospitals NHS Foundation Trust, Cambridge, UK
7. NIHR Respiratory Biomedical Research Unit, Royal Brompton & Harefield NHS Foundation Trust and Imperial College, London, UK

*joint first authors

**Corresponding author:** Marie Fisk, Division of Experimental Medicine & Immunotherapeutics, Level 3, Box 98, Addenbrookes Hospital, Hills Road, CB2 0QQ. Telephone: +44 (0) 1223336806. Email: [mf503@medschl.cam.ac.uk](mailto:mf503@medschl.cam.ac.uk)

**Expanded methods:**

**Vascular biomarkers and laboratory assessments:**

All studies were conducted in a quiet, temperature-controlled room. In all individuals, brachial blood pressure was recorded after 15-minutes of rest (seated prior to augmentation index (AIx) measurement and supine prior to aortic pulse wave velocity measurement (aPWV)), using a validated oscillometric device (HEM 750CP; Omron Corporation, Japan). Blood pressure was measured in the non-dominant arm and three measurements taken, with a 1-minute interval between them. The average of the 2^nd^ and 3^rd^ reading was recorded and used in analysis. If the difference between the 2^nd^ and 3^rd^ exceeded 5mm Hg, further readings were taken. Assessments of vascular biomarkers (AIx, aPWV and carotid intima media thickness (CIMT)) for chronic obstructive pulmonary disease (COPD) patients were performed after a 12-hour period without inhaled medications, with the exception of short acting beta agonist use allowed no less than 6-hours before assessments if needed for symptomatic relief.

Trained investigators performed all vascular assessments in duplicate, and mean values were used in subsequent analysis. The within- and between-observer measurement reproducibility values were in agreement with our previously published data.^1,2^

Serum samples were analyzed in accredited laboratories. The estimated glomerular filtration rate (eGFR) was calculated as per the Modified Diet and Renal Disease equation.^3^

**COPD severity:**

The BODE Index score (0-10) is a multidimensional COPD severity classification, comprising of four weighted variables: **B**ody mass index (kg/m^2^), **O**bstruction (Forced Expiratory Lung Volume in 1 second % predicted), **D**yspnea (modified MRC score (0-4)), and **E**xercise capacity (6-minute walk distance (metres)) that robustly predicts mortality in COPD.^4^ COPD patients in the study were stratified into BODE quartiles (Q1: 0-2, Q2: 3-4, Q3: 5-6, Q4: 7-10), to evaluate for differences in aPWV, AIx and CIMT across quartiles. Additionally, the relationship between reported exacerbations (defined as worsening respiratory symptoms requiring treatment with antibiotics and/or steroids) and these vascular biomarkers was examined.

**References:**

1. Mohan D, Gale NS, McEniery CM, Bolton CE, Cockcroft JR, MacNee W, Fuld J, Lomas DA, Calverley PMA, Shale DJ, Miller BE, Wilkinson IB, Tal-Singer R, Polkey MI, ERICA Consortium. Evaluating the role of inflammation in chronic airways disease: the ERICA study. *COPD.* 2014;11:552-559. doi:10.3109/15412555.2014.898031.

2. Wilkinson IB, Fuchs SA, Jansen IM, Spratt JC, Murray GD, Cockcroft JR, Webb DJ. Reproducibility of pulse wave velocity and augmentation index measured by pulse wave analysis. *J Hypertens.* 1998;16:2079-2084.

3. Levey AS, Bosch JP, Lewis JB, Greene T, Rogers N, Roth D. A More Accurate Method To Estimate Glomerular Filtration Rate from Serum Creatinine: A New Prediction Equation*. Ann Intern Med.* 1999;130:461-470. doi:10.7326/0003-4819-130-6-199903160-00002.

4. Celli BR, Cote CG, Marin JM, Casanova C. The body-mass index, airflow obstruction, dyspnea, and exercise capacity index in chronic obstructive pulmonary disease. *N Engl J Med.* 2004;350:1005-1012. doi:10.1056/NEJMoa021322.

**Table S1: Demographic and haemodynamic characteristics of COPD patients and controls without known cardiovascular comorbidity.**

Data are means±(standard deviation (SD)) or percentage. Subjects with any known cardiovascular comorbidity (angina, myocardial infarction, stroke, peripheral vascular disease, diabetes, anti-hypertensive treatment, cholesterol-reducing therapy) were excluded from analysis. BP=blood pressure, PWV= pulse wave velocity, IMT=intima media thickness. *Aortic pulse wave velocity adjusted for gender, heart rate, mean arterial pressure and study site. ^†^Augmentation index adjusted for gender, height, heart rate and study site. ‡Carotid intima media thickness adjusted for systolic blood pressure and study site.

| **Variable** | **COPD**  **n=190** | **Controls**  **n=660** | **p-value** |
| --- | --- | --- | --- |
| Age (years) | 66±8 | 65±9 | 0.8 |
| Male n (%) | 100 | 405 | 0.02 |
| Body mass index (kg/m^2^) | 26.08±5.78 | 26.55±3.89 | 0.2 |
| Systolic BP (mmHg) | 143±19 | 140±18 | <0.001 |
| Diastolic BP (mmHg) | 81±10 | 82±10 | 0.7 |
| Mean arterial pressure (mmHg) | 96±11 | 96±11 | 0.8 |
| Pulse pressure (mmHg) | 62±16 | 56±14 | <0.001 |
| Heart rate (bpm) | 75±12 | 71±12 | <0.001 |
| Adjusted Aortic PWV (m/s)* | 9.48±2.34 | 8.87±2.06 | 0.003 |
| Adjusted Augmentation Index (%)^†^ | 28±9 | 24±8 | <0.001 |
| Carotid IMT (mm)‡ | 0.79±0.20 | 0.73±0.20 | 0.007 |

**Table S2: Demographic and haemodynamic characteristics in COPD patients and controls stratified by smoking status**

| **Variable** | **Never-Smoker** | **Ex-Smoker** | **Smoker** | **p-value** |
| --- | --- | --- | --- | --- |
| **Controls** | | | | |
| Number (%) | 939 (57%) | 647 (39%) | 71 (4%) | - |
| Age (years) | 68±8 | 68±8 | 65±10 | 0.01 |
| Male n (%) | 476 (51%) | 443 (68%) | 59 (83%) | <0.001 |
| BMI (kg/m^2^) | 27.00±4.42 | 27.95±4.21 | 26.75±3.41 | <0.001 |
| FEV_1_ (L) | 2.47±0.83 | 2.52±0.84 | 2.62±0.80 | 0.3 |
| FEV_1_ (%) | 102±17 | 98±18 | 92±17 | <0.001 |
| HR (bpm) | 71±12 | 70±12 | 73±12 | 0.1 |
| SBP (mmHg) | 140±18 | 141±17 | 138±21 | 0.1 |
| DBP (mmHg) | 80±10 | 82±10 | 81±11 | 0.08 |
| MAP (mmHg) | 96±11 | 97±10 | 95±12 | 0.006 |
| aPWV (m/s)* | 9.22±1.87 | 9.04±1.89 | 9.49±1.86 | 0.07 |
| AIx (%)^†^ | 25±7 | 25±07 | 26±7 | 0.07 |
| **COPD patients** | | | | |
| Number (%) | 0 | 307 (67%) | 151 (33%) | - |
| Age (years) | - | 69±8 | 64±8 | <0.001 |
| Male n (%) | - | 181 (59%) | 83 (55%) | 0.2 |
| BMI (kg/m^2^) | - | 28.09±5.48 | 26.87±6.07 | 0.04 |
| FEV_1_ (L) | - | 1.31±0.51 | 1.39±0.56 | 0.1 |
| FEV_1_ (%) | - | 53±16 | 53±16 | 0.8 |
| HR (bpm) | **-** | 74±12 | 75±12 | 0.53 |
| SBP (mmHg) | **-** | 147±18 | 143±19 | 0.03 |
| DBP (mmHg) | **-** | 82±11 | 81±11 | 0.8 |
| MAP (mmHg) |  | 97±11 | 96±11 | 0.09 |
| aPWV (m/s)* | **-** | 10.34±2.07 | 10.26±1.76 | 0.7 |
| AIx (%)^†^ | **-** | 27±7 | 29±8 | 0.02 |
| CIMT‡ (mm) | **-** | 0.86±0.17 | 0.85±0.18 | 0.6 |

Data are means±SD or percentage. BMI=body mass index, HR= heart rate, SBP=systolic blood pressure, DBP=diastolic blood pressure, MAP=mean arterial pressure, aPWV= aortic pulse wave velocity, AIx= augmentation index. *Aortic pulse wave velocity adjusted for age, gender, body mass index, heart rate, mean arterial pressure and study site. ^†^Augmentation index adjusted for age, gender, height, heart rate and study site. ‡Carotid intima media thickness adjusted for age, systolic blood pressure and study site.

**Table S3: Demographic and vascular characteristics of COPD patients classified by BODE Index score quartiles**

| **Variable** | | | **Q1**  **0-2**  **n=197** | **Q2**  **3-4**  **n=95** | **Q3**  **5-6**  **n=87** | **Q4**  **7-10**  **n=48** | **p-value** |
| --- | --- | --- | --- | --- | --- | --- | --- |
| Age (years) | | | 67±8 | 67±8 | 67±8 | 67±8 | 0.8 |
| Male n (%) | | | 125 (63%) | 45 (47%) | 50 (57%) | 29 (60%) | 0.07 |
| BMI (kg/m^2^) | | | 27.62±4.19 | 27.60±5.48 | 28.08±6.72 | 26.68±7.64 | 0.6 |
| Current smoker n (%) | | | 60 (30%) | 33 (35%) | 33 (38%) | 16 (33%) | 0.7 |
| Ex-smoker n (%) | | | 137 (70%) | 62 (65%) | 54 (62%) | 32 (67%) |
| Total pack years | | | 47±26 | 48±31 | 46±25 | 54±27 | 0.4 |
| Oxygen saturations (%) | | | 97±2 | 96±2 | 95±3 | 96±2 | <0.001 |
|  |  | **Respiratory Medication** | | | | | |
| ICS+LABA (%) | | | 49% | 62% | 71% | 79% | <0.001 |
| ICS (%) | | | 7% | 8% | 8% | 6% | 0.9 |
| LABA (%) | | | 7% | 10% | 10% | 8% | 0.8 |
| LAMA (%) | | | 49% | 72% | 76% | 75% | <0.001 |
|  |  | **Cardiovascular medical history** | | | | | |
| Angina (%) | | | 6% | 19% | 6% | 13% | 0.02 |
| Myocardial infarction (%) | | | 5% | 12% | 6% | 10% | 0.2 |
| Stroke (%) | | | 9% | 10% | 6% | 7% | 0.7 |
| Diabetes (%) | | | 12% | 19% | 11% | 15% | 0.5 |
| PVD (%) | | | 7% | 8% | 1% | 6% | 0.2 |
| Cholesterol therapy (%) | | | 33% | 47% | 33% | 30% | 0.1 |
| Anti-hypertensive therapy (%) | | | 39% | 39% | 32% | 36% | 0.7 |
|  |  | **Haemodynamic assessment** | | | | | |
| SBP (mmHg) | | | 146±18 | 142±17 | 148±20 | 140±19 | 0.02 |
| DBP (mmHg) | | | 82±10 | 81±11 | 83±11 | 78±13 | 0.08 |
| MAP (mmHg) | | | 103±11 | 101±11 | 104±12 | 98±13 | 0.03 |
| Pulse pressure (mmHg) | | | 65±16 | 64±14 | 63±14 | 64±16 | 0.9 |
| Heart rate (bpm) | | | 68±11 | 71±10 | 71±12 | 76±11 | <0.001 |
| aPWV (m/s) | | | 9.99±2.52 | 10.17±2.22 | 10.47±2.42 | 11.37±2.79 | <0.001 |
| aPWV (m/s)* | | | 10.01±2.46 | 10.21±2.36 | 10.37±2.38 | 11.39±2.43 | 0.01 |
| Augmentation index (%) | | | 28+10 | 28±10 | 28±11 | 24±9 | 0.1 |
| Augmentation index (%)^†^ | | | 27±7 | 27±7 | 28±7 | 27±7 | 0.8 |
| Carotid IMT (mm) | | | 0.86±0.18 | 0.86±20 | 0.83±18 | 0.84±0.21 | 0.6 |
| Carotid IMT (mm)‡ | | | 0.86±0.19 | 0.86±0.19 | 0.87±0.18 | 0.84±0.19 | 0.5 |

Data are means±SD or percentage. BMI=Body mass index, ICS+LABA=Combined inhaled corticosteroid and long acting beta agonist inhaler, ICS=inhaled corticosteroid inhaler, LABA=long acting beta agonist inhaler, LAMA=long acting muscarinic antagonist, SBP=systolic blood pressure, DBP=diastolic blood pressure, MAP=mean arterial pressure, aPWV=aortic pulse wave velocity, IMT=intima media thickness. *adjusted for heart rate, mean arterial pressure, study site, LAMA use, ICS+LABA use and oxygen saturations ^†^=adjusted for heart rate, height and study site, ‡=adjusted for study site.

**Table S4: BODE Index association with aortic pulse wave velocity in COPD patients**

| **Aortic pulse wave velocity** | **Beta** | **p-value** |
| --- | --- | --- |
| Adjusted R^2^=0.41, P<0.001 |  |  |
| Age (years) | 0.46 | <0.001 |
| Mean arterial pressure (mmHg) | 0.21 | <0.001 |
| Heart rate (bpm) | 0.16 | <0.001 |
| Glucose (mmol/L) | 0.12 | 0.006 |
| BODE Index (0-10) | 0.10 | 0.01 |
| PVD (yes/no) | 0.09 | 0.03 |
| Body mass index (kg/m^2^) | 0.09 | 0.04 |

PVD=Peripheral vascular disease. Variables that were not independently associated with aPWV: cholesterol reducing therapy (yes/no), total cholesterol (mmol/L), triglycerides (mmol/L), smoker (yes/no), total pack years smoked, diabetes (yes/no), angina (yes/no), myocardial infarction (yes/no), stroke (yes/no), anti-hypertensive therapy (yes/no).

**
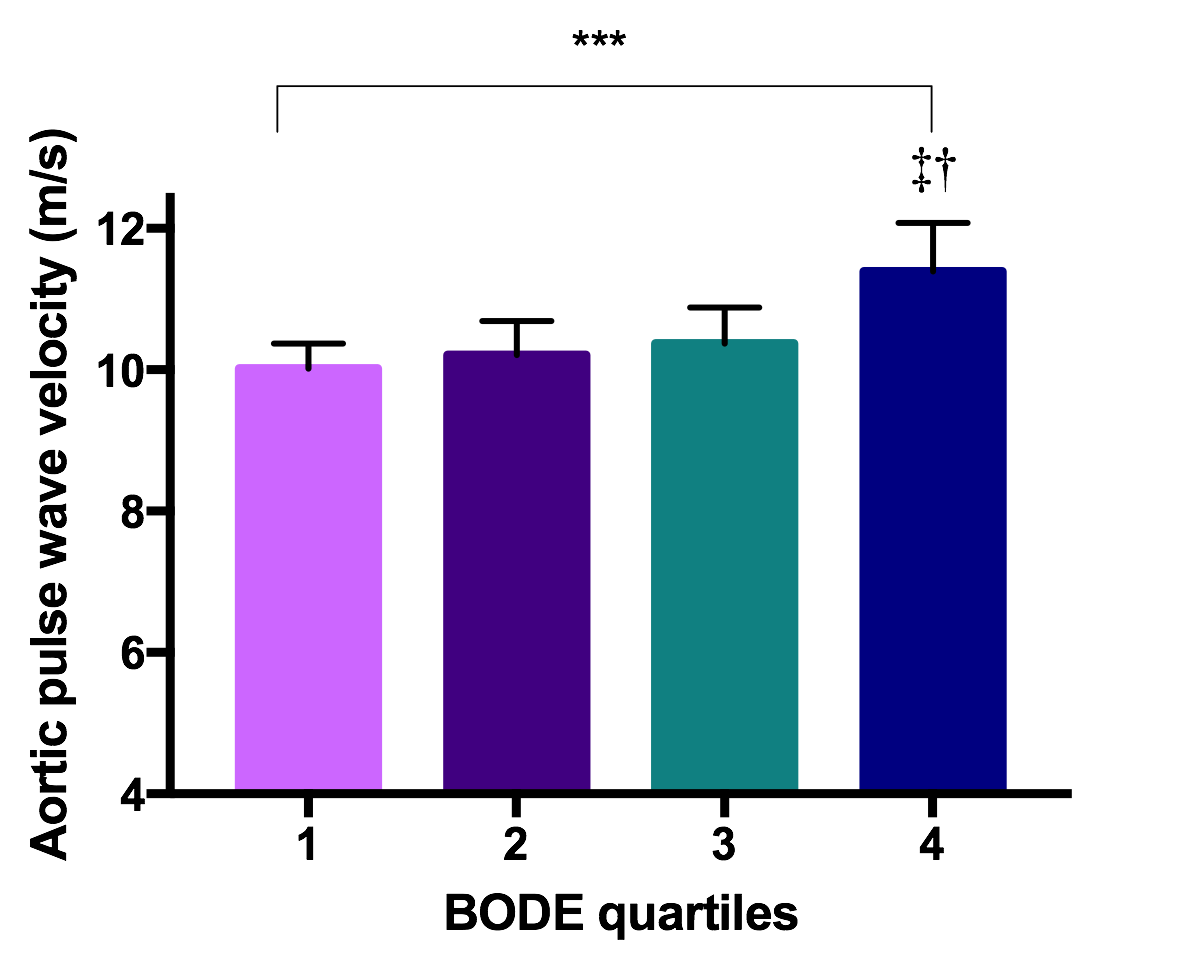
**

**Figure S1: Aortic pulse wave velocity stratified by BODE Index quartiles of COPD patients.**

***P<0.001 4^th^ vs 1^st^ quartile, †P=0.006 4^th^ vs 2^nd^ quartile, ‡P=0.02 4^th^ vs 3^rd^ quartile. Bars represent mean values, error bars represent 95% confidence intervals. Aortic

pulse wave velocity adjusted for heart rate, mean arterial pressure, study site, long acting muscarinic antagonist use, combined long acting beta agonist/inhaled corticosteroid use, peripheral oxygen saturations.
