# Supplementary figures and images for: Surrogate Markers of Cardiovascular Risk and Chronic Obstructive Pulmonary Disease: A Large Case-Controlled Study
Source: Hypertension. 2018 Aug 20;71(3):499–506. doi: 10.1161/HYPERTENSIONAHA.117.10151 (PMC5805278; doi:10.1161/HYPERTENSIONAHA.117.10151)

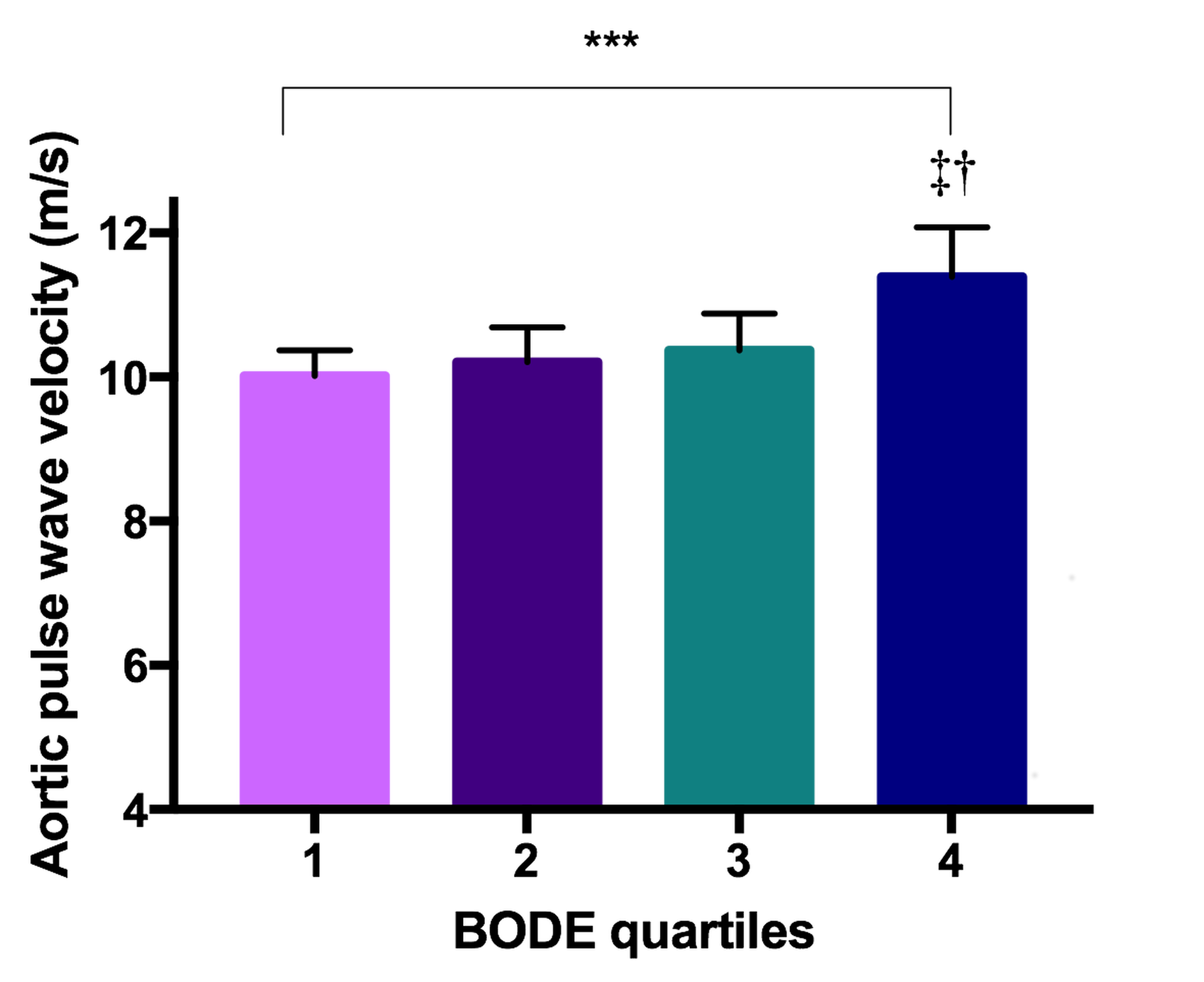

Supplement: Supplementary file 2 [file hyp-71-499-s002.tif]
